# Vitamin D status in chronic dialysis patients with depression: a prospective study

**DOI:** 10.1186/1471-244X-14-125

**Published:** 2014-04-28

**Authors:** Jisheng Zhang, Ping Zhang, Xiaoying Ni, Beiyan Bao, Congyang Huang, Yongyao Wu, Min Ni, Jinfeng Duan, Jianghua Chen

**Affiliations:** 1 Department of Nephrology, Beilun Branch of the First Affiliated Hospital, College of Medicine, Zhejiang University, Ningbo, Zhejiang, PR China; 2 Department of Nephrology, the First Affiliated Hospital, College of Medicine, Zhejiang University, Hangzhou, Zhejiang, PR China; 3 Department of Neurology, People's Hospital of Yinzhou, College of Medicine, Ningbo University, Ningbo, Zhejiang, PR China; 4 Department of Nephrology, Ningbo Urology and Nephrology Hospital, College of Medicine, Ningbo University, Ningbo, Zhejiang, PR China; 5 Department of Psychiatry, Beilun Branch of the First Affiliated Hospital, College of Medicine, Zhejiang University, Ningbo, Zhejiang, PR China; 6 Department of Psychiatry, the First Affiliated Hospital, College of Medicine, Zhejiang University, Hangzhou, Zhejiang, PR China

**Keywords:** Depression, Vitamin D, End-stage renal disease, Dialysis, Efficacy, Prospective study

## Abstract

**Background:**

Depression is the most widely acknowledged psychological problem among end-stage renal disease (ESRD) patients. Depression may be associated with VD deficiency. The aims of this study are to (a) elucidate the prospective association between HsCRP, VD contents and depressive symptoms in the dialyzed population, and (b) find the effect of calcitriol supplementation on depression in dialyzed patients.

**Methods:**

In this prospective study, 484 dialysis patients (382 hemodialysis [HD] cases and 102 peritoneal dialysis [PD] cases; aged 18–60 years) from two hospitals in southeast China were included. The depression in these patients was evaluated using the Chinese version of Beck’s Depression Inventory (BDI). All subjects answered the BDI-I questionnaire for assessment of depression levels in summer. A cut-off value of 16 was set to include dialysis patients with depression. All patients were divided into two groups depending on the absence (Group1) or presence (Group 2) of depression. The two groups took 0.5 μg/day 1,25-Dihydroxyvitamin D orally for one year. BDI Scores were recalculated for all patients. Sociodemographic, clinical data, and serum VD contents were also collected.

**Results:**

A total of 484 participants (247 men [51.0%] and 237 women [49.0%]) were surveyed. Depressive symptoms were found in 213 (44.0%) patients. The baseline serum VD level (VD2 + VD3) was 17.6 ± 7.7 nmol/L. Patients with depressive symptoms have significantly higher serum HsCRP level and significantly lower serum VD level compared with the control group. After one-year follow-up, the supplementation of 0.5 μg/day calcitriol slightly improved the microinflammatory state such as lowering mean serum HsCRP level and improving serum VD level, but not in significantly enhancing the depressive symptoms.

**Conclusions:**

Calcitriol supplementation did not significantly enhance the depressive symptoms in our dialyzed population although patients with low levels of serum VD were more depressed. Therefore, more prospective randomized controlled trials are necessary to reveal the exact cause-and-effect relationship between VD status and depressive symptoms or VD status related to some specific subtypes in dialyzed patients.

## Background

Depression ranks fourth on the World Health Organization (WHO) global disease burden list. Depression is the most widely acknowledged psychological problem among end-stage renal disease (ESRD) patients [1,2]. About 28% of chronic kidney disease patients facing impending dialysis undergo major depression, and even a larger proportion of dialyzed patients suffer from depression [3]. Inflammation in the body is a common manifestation to many diseases, including high blood pressure, coronary artery disease, diabetes as well as chronic kidney disease [4,5]. Recently, many authors have tried to reveal the connection between depression and inflammatory status in ESRD patients [6,7]. Depression is also linked to an inflammation marker in blood called C-reactive protein (CRP). Proinflammatory cytokines were proven very important in the pathogenesis of depression in the general population and the levels of the same cytokines elevated in ESRD patients [8]. However, repeated episodes of depression contribute to inflammation in the body, which highlights a potentially important role for untreated depression as a contributor to a series of serious medical problems [9]. The causality between depression and inflammation is still unclear.

Vitamin D (VD) is an essential nutrient for bone health, and has other physiological functions. There are plausible reasons for investigating the curative effects of VD for depressive disorders. Depression may be associated with VD deficiency [10-12]. Hypovitaminosis D and secondary hyperparathyroidism commonly occur in the dialyzed population for several reasons [13,14]. Therefore, Calcitriol supplementation is popular among dialyzed patients. However, spontaneous 25(OH)D levels > 20 ng/mL seem sufficient to control serum intact parathyroid hormone (iPTH) in chronic kidney disease (CKD) patients [15]. These findings reinforce the guidelines to supplement VD only if less than 30 ng/mL. In China, Calcitriol is used as a form of VD to treat hypovitaminosis D and secondary hyperparathyroidism in patients whose kidneys or parathyroid glands are not working normally.

However, it is unknown whether serum VD content is associated with depression in the dialyzed population and whether calcitriol supplementation is efficient for treatment of depression. The intentions of this study were to (a) investigate the prospective association between HsCRP, VD contents and depressive symptoms in the dialyzed population, and (b) find the efficacy of calcitriol supplementation on depression in dialyzed patients.

## Methods

### Participant selection

In this prospective study, we included a total of 484 patients receiving dialysis (382 hemodialysis [HD] cases and 102 peritoneal dialysis [PD] cases) from two hospitals in China. All patients aged 18–60 who had received HD or PD for more than 3 months were screened for participation. Signed informed consent was required for enrollment, and after that, patients received oral and written information about the study. Ethical permission for the study was obtained from the Scientific Ethical Committee in Beilun Branch of the First Affiliated Hospital of Zhejiang University (Approval No. 2012033).

### Exclusion criteria

Exclusion criteria: 1, cognitive deficits such as considerable memory loss, confusion/dementia, and intellectual disability; illiteracy and/or incapability of answering the questionnaire (difficulty in understanding the questions, visual or hearing impairment); 2, Evidence shows that the incidence of depression may increase in older patients compared with normal adults [16]. Thus, the patients more than 60 years old were excluded from this study; 3, Patients used antidepressant medications in recent two years; 4, Patients had severe depressive symptoms before dialysis.

### Depressive symptoms and division

Depression is a condition characterized by depressed mood or loss of interest or pleasure in nearly all activities almost every day for at least 2 weeks. The presence of depressive symptoms was determined using a Chinese version of Beck’s Depression Inventory (BDI) in summer, and the Chinese version was previously validated as the English version [17-20]. The BDI consists of 21 self-reported items and each item is rated on the scale of 0–3, producing a possible score range from 0 to 63. A higher score correlates with more severe depression [21]. BDI score >16 is characteristic for presence of moderate to severe depressive symptoms. A cut-off value of 16 was set to include the dialyzed patients with depression [22,23]. BDI score ≥ 30 was defined as severe depression symptoms [24,25]. Structured interview was conducted to determine diagnoses and severity of depression for each patient by two experienced psychiatrist independently.

All patients were divided into two groups depending on the absence or presence of depression. In China, dialysis patients usually took 1,25- Dihydroxyvitamin D (Calcitriol, Roche, Shanghai, China) orally 0.25 - 0.5 μg/day or 1.25 μg - 2.5 μg twice a week. Therefore, the two groups took 0.5 μg/day calcitriol orally for one year, and BDI scores were recalculated.

### Demographic and clinical data at baseline

Demographic data including employment status and education status were collected by questioning the patients. Information concerning age, sex, primary kidney disease, dialysis modality, comorbidities, health insurance, and mean time on dialysis (months) was gathered from medical records. Comorbidity was scored on the number of comorbid conditions using the comorbidity index according to a previous study [26].

Blood was collected according to a standard protocol. Laboratory data including levels of intact parathyroid hormone (iPTH), serum albumin, high sensitive C-reactive protein (hsCRP) and serum calcium were gathered from medical records. Serum VD (VD2+ VD3) levels were analyzed using liquid chromatography–mass spectrometry (LC-MS, or the gold standard high-performance liquid chromatography [HPLC]-MS [27,28]) at the Kingmed Diagnostics Laboratory, Guangzhou, China.

25-hydroxyvitamin D2 and 25-hydroxyvitamin D3 in serum were detected with HPLC-MS according to a previous study [29]. Human serum (150 μL) was diluted in 450 μL of 2-propanol containing butylated hydroxytolouene as an antioxidant. After thorough mixing (15 min) and centrifugation (10 min, 4000 g at 10°C), an aliquot of 35 μL was injected from the supernatant into the HPLC system. HPLC was performed with an HP 1100 liquid chromatograph interfaced by atmospheric pressure chemical ionisation to an HP mass spectrometric detector operated in the single ion monitoring mode. VD analogues were separated on a 4.6 mm × 50 mm reversed phase column with 1.8 μM particles. The column temperature was 80°C. A two-point calibration curve was plotted from analysis of an albumin solution with known VD concentration.

### Statistical analysis

We calculated and proved that a sample of 484 patients provided adequate power (α = 0.05 in two-sided t-test) for the proposed tests in this two-group study. Changes in HsCRP, VD contents and BDI scores from the baseline to the one-year follow-up were presented in a figure with 2 panels of the mean and error bars of each variable. The results were expressed as mean ± standard deviation (SD) and P < 0.05 was considered significant. The prevalence of depression was evaluated using frequency analysis and 95% confidence intervals (CIs). Differences in means were tested using an independent t-test, or Mann–Whitney test. Quartiles were compared with one-way analysis of variance (ANOVA), Kruskal–Wallis test, or χ^2^ test as appropriate. The effects of the VitD treatment were examined using 2x2 ANOVA.

The independent risk factors associated with BDI scores in dialysis patients were defined using multiple linear regression analysis. Depression can be affected by many factors including sex, marital stage, employment status, health insurance, living, comorbidity index, dialysis modality, hemoglobin, serum albumin, sensitive C-reactive protein, and VD. Therefore, all analyses were adjusted for those variables. All statistical analyses were evaluated using SPSS 17.00 (IBM Corporation, Armonk, NY, USA).

## Results

### Baseline characteristics and assessment of depressive symptoms in dialyzed patients

The 484 respondents were aged 52.7 ± 15.9 years on average. Their general characteristics are presented in Table 1. There were some differences in age, sex, comorbidity index, mean hemoglobin, mean sensitive C-reactive protein, and mean serum VD.

**Table 1:** Socialdemographic and clinical datas between depression and non-depression groups

**Characteristics**	**Group1 (BDI score 0 ~ 16) (n = 271)**	**Group2 (BDI score > 16) (n = 213)**	**P value**
Age (Yr)	49.7 ± 19.0	56.5 ± 12.0	0.03
Female sex. No. (%)	99 (36.5)	138 (64.8)	<0.001
Dialysis modality (haemodialysis, %)	209 (72.1)	173 (82.1)	0.32
Marital status. No. (married, %)	221 (81.5)	165 (77.5)	0.32
Employment status (Employed)	180 (66.4)	134 (62.9)	0.48
Primary kidney disease. No. (%)			0.41
Nephritis	149 (55.0)	126 (59.1)	
Hyptertensive nephrosclerosis	14 (5.2)	10 (4.7)	
Diabetic nephrology	18 (6.6)	44 (20.7)	
Polycystic kidney disease	17 (6.3)	13 (6.1)	
Other	73 (26.9)	19 (9.4)	
Health insurance modes. No. (%)			0.61
The medical insurance	210 (77.5)	160 (75.1)	
The new rural insurance	61 (22.5)	53 (24.9)	
Education			0.09
UP to high school	55 (20.3)	58 (27.2)	
Beyond high school	216 (79.7)	155 (72.8)	
living along	7 (2.6)	13 (6.1)	0.08
Mean hemoglobin (g/l) (mean ± SD)	12.4 ± 5.7	10.8 ± 1.7	0.04
Mean serum calcium (g/l) (mean ± SD)	2.4 ± 0.6	2.2 ± 0.9	0.57
Mean serum albumin (g/l) (mean ± SD)	35.3 ± 6.8	34.3 ± 2.7	0.63
Serum vitamin D (nmol/l) (mean ± SD)	19.3 ± 9.7	15.4 ± 5.1	0.032
Plasm iPTH (pmol/l) (mean ± SD)	213.6 ± 89.4	197.4 ± 103.5	0.08
High sensitive C-reactive protein (mg/l) (mean ± SD)	7.8 ± 2.6	9.7 ± 3.3	0.014
Mean time on dialysis (months) (mean ± SD)	56.5 ± 12.2	47.6 ± 16.2	0.42
Comorbidity index (mean ± SD)	5.5 ± 0.3	7.8 ± 3.1	0.007

Differences in proportions were tested using Pearson chi-square test; differences in means were tested using an Independent t-test, or Mann–Whitney.

*Abbreviation*: *n* number, *iPTH* intact parathyroid hormone.

The baseline serum VD (VD2+ VD3) level of the dialyzed population was 11.9 ± 6.8 (3.6 to 32.5) nmol/L. Overall BDI score for the studied population was 17.3 ± 7.2 points. Moderate to severe depressive symptoms (BDI score > 16) were found in 213 (44.0%, 95% CI: −0.137-0.027) patients. The depression group has significantly higher serum HsCRP level (9.7 ± 3.3 vs. 7.8 ± 2.6 mg/l; p = 0.014) and significantly lower serum VD level (15.4 ± 5.1 vs. 19.3 ± 9.7 mg/l; p = 0.032) compared with the non-depression group.

### Risk factors for depression in the dialyzed patients

The independent risk factors associated with BDI score in dialysis patients were defined using multiple linear regression analysis. The independent variables such as age, sex, marital stage, employment status, health insurance, living, comorbidity index, and dialysis modality were inserted in the analysis with the BDI scores as criterion. Only sex and comorbidity index were independently associated with BDI scores, indicating that (1) The females were more likely to have higher BDI scores compared with males; (2) the dialysis patients with more comorbid conditions were more likely to have higher BDI scores compared with those with less comorbid conditions; (3) Patients with higher BDI scores have significantly higher serum HsCRP level and significantly lower serum VD level, not vice-versa.

### Efficacy of calcitriol supplementation on treatment of depression in dialyzed patients

**Figure 1:**
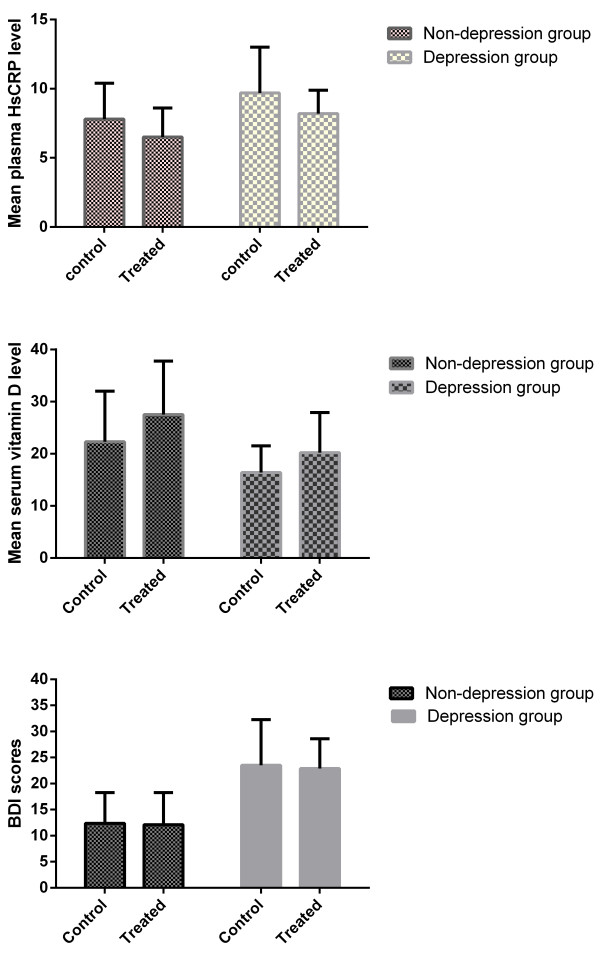
**Mean serum HsCRP, serum vitamin D level and BDI scores between groups after one year follow up.** HsCRP, hypersensitive C reactive protein. Mean serum HsCRP in mg/l and serum vitamin D level in nmol/l. BDI, Beck Depression Inventory, with the score ranges from 1 to 63, with higher score indicating more severe depressive symptoms. Control, before treatment; treated, after treatment.

Data on serum HsCRP level, VD (VD2+ VD3) levels, and BDI scores after one year follow-up are listed in Figure 1. Calcitriol supplementation slightly improved the microinflammatory state such as lowering mean serum HsCRP level (8.2 ± 1.7 vs. 9.7 ± 3.3, P = 0.027/6.5 ± 2.1 vs. 7.8 ± 2.6, P = 0.042), but associations did not substantially change when depression was included in the model in dialysis population.

## Discussion

This prospective study provided a comprehensive and detailed description of the incidence of depression and the effect of calcitriol supplementation on treatment of depression in dialyzed patients. The overall incidence of depression was (44%, 95% CI: −0.137-0.027) in the dialyzed patients, which was consistent with previous reports [30-32]. The high occurrence of depression may be partly due to anemia and poor nutritional status in the dialyzed population [33]. Moreover, employment status, health insurance mode, marital status, and constipation status can all affect the incidence of depression. In our study, anemia (hemoglobin level) and nutritional status (albumin) were also evaluated, and there was difference in mean hemoglobin level, but not in mean albumin level. Thus, we thought depression for dialysis patients was multifactorial apart from poor nutritional status.

The depressive patients have higher serum HsCRP level than the non- depressive patients, and thus CRP may be as a new risk factor of depression in the dialyzed population. Also HsCRP level can be used to predict the outcome in HD patients. After one-year follow up, calcitriol supplementation slightly improved the level of mean serum HsCRP, which was not accompanied by the improvement of depressive symptoms. The causality between depression and inflammation is not well clear. Depression was linked to worse nutritional status in ESRD patients, [34] indicating that repeated episodes of depression contribute to inflammation, rather than inflammation contribute to depression in the body [9].

We did find an association between depressive symptoms and serum VD (VD2+ VD3) levels in the dialyzed population. The patients with higher serum VD levels at baseline tended to have fewer depressive symptoms. Recently, the possible link between depression and VD status has been investigated in several small studies, but the findings are inconsistent because of differences in races, study populations, geographic locations and methodologies [35-39]. In particular, the depressive state may be a cause rather than a consequence of VD deficiency, because if depression caused VD deficiency owing to lack of sunlight exposure, then the association between VD levels and depressive symptoms would be stronger in the summer months when VD levels are most strongly influenced, which was not evident in this sample. Serum VD level is related to physical activity (time spent outdoors), body mass index (BMI), chronic diseases, social status and nutritional status. Luigi Ferrucci et al. explored whether low VD levels and depression in older people were related [40]. They did not find that low vitamin D levels can cause depression, and thus they thought other characteristics might predispose people with low nutrient levels to depression [40]. Also the most important one probably is that people with low VD are likely to have all these pathological problems associated with low VD and these problems can cause depression. Therefore, prospective research is required to clarify the direction of cause and effect [40].

The effect of VD in the brain is not well understood. VD is considered as a neurosteroid across the blood–brain barrier. The VD receptors widespread in the brain include the hippocampus, which is associated with the development of depression. In our intervention, we did not find a significant effect of calcitriol supplementation on enhancing the depressive symptoms in the dialyzed population, since the effects are also conflicting [41-43]. There are some reasons for this result. First, calcitriol supplementation did not significantly enhance the depressive symptoms in the dialyzed population since we did not find the correct cause-and-effect relationship between VD and depression. Second, some causes lead to the opposite result: (1) The tested dose of VD (0.5 μg/day) might be insufficient to affect the occurrence of depression, and the VD intake from food sources was ignored. (2) One year was a rather short time frame for investigation of depression, since depression may develop slowly and last for several years. (3) BDI scores reflect the self-report of depressive symptoms, but it was controversial that a cut-off value of 16 was set to include the patients with depression (Some study chosen BDI score ≥ 20 as moderate to severe depression) [44]. It stressed that calcitriol supplementation can improve cardiovascular symptoms, [45,46] and reduce vascular depression, but many reasons may induce the occurrence of depression in dialyzed population. Additionally, we use the dialyzed patients as objects for the first time.

A few limitations in the current study should be noted. Specifically, although we assessed a broad range of depressive disorders, it is difficult to ascertain the specific causes of depression within the sample. Second, We did not subdivide the patients with depression: one group treated with the active compound and one treated with placebo. It was not a randomized double-blind trial study. Thirdly, the dose of 0.5 μg calcitriol for dialyzed patients is far enough for curing depression. Moreover, the sample in our study is far smaller than enough.

## Conclusions

Even though people with low levels of serum VD were more depressed, Calcitriol supplementation did not significantly enhance the depressive symptoms in our dialysis population. More prospective randomized controlled trials are necessary to find the exact cause-and-effect relationship between VD status and depressive symptoms or VD status related to some specific subtypes in dialyzed patients.

## Competing interests

The authors report no conflicts of interest. The authors alone were responsible for the content and writing of the paper.

## Authors’ contributions

JZ, BB and CH carried out the literature study, data collection, data analysis and drafted the manuscript. The presence of depressive symptoms among patients was determined using a Chinese version of Beck’s Depression Inventory (BDI) by MN and JD. PZ and JC critically revised the manuscript. XN and YW performed data analysis and critically revised the manuscript. All authors read and approved the final manuscript.

## Pre-publication history

The pre-publication history for this paper can be accessed here:

http://www.biomedcentral.com/1471-244X/14/125/prepub

## References

[B1] Ibrahim N Chiew-Thong NK Desa A Razali R Depression and coping in adults undergoing dialysis for end-stage renal disease Asia-Pacific Psychiatry 2013 5 Suppl 1 35 40 23857835 10.1111/appy.12042

[B2] McKercher C Sanderson K Jose MD Psychosocial factors in people with chronic kidney disease prior to renal replacement therapy Nephrology (Carlton, Vic) 2013 18 9 585 591 10.1111/nep.12138 23876102

[B3] Park HC Lee H Lee JP Kim DK Oh KH Joo KW Lim CS Kim YS Ahn C Oh YK Lower residual renal function is a risk factor for depression and impaired health-related quality of life in Korean peritoneal dialysis patients J Korean Med Sci 2012 27 1 64 71 10.3346/jkms.2012.27.1.64 22219616 PMC3247777

[B4] Hung AM Ikizler TA Griffin MR Glenn K Greevy RA Grijalva CG Siew ED Crawford DC CRP polymorphisms and chronic kidney disease in the third national health and nutrition examination survey BMC Med Genet 2011 12 65 21569369 10.1186/1471-2350-12-65 PMC3119179

[B5] Hung AM Crawford DC Griffin MR Brown-Gentry K Lipkowitz MS Siew ED Cavanaugh K Lewis JB Ikizler TA CRP polymorphisms and progression of chronic kidney disease in African Americans Clin J Am Soc Nephrol 2010 5 1 24 33 10.2215/CJN.01900309 19965533 PMC2801650

[B6] Li ZJ An X Mao HP Wei X Chen JH Yang X Zhou SF Li ZB Yu XQ Association between depression and malnutrition-inflammation complex syndrome in patients with continuous ambulatory peritoneal dialysis Int Urol Nephrol 2011 43 3 875 882 10.1007/s11255-011-9917-x 21360161

[B7] Czira ME Lindner AV Szeifert L Molnar MZ Fornadi K Kelemen A Laszlo G Mucsi I Keszei AP Kennedy SH Novak M Association between the Malnutrition-Inflammation Score and depressive symptoms in kidney transplanted patients Gen Hosp Psychiatry 2011 33 2 157 165 10.1016/j.genhosppsych.2011.01.012 21596209

[B8] Stenvinkel P Barany P Heimburger O Pecoits-Filho R Lindholm B Mortality, malnutrition, and atherosclerosis in ESRD: what is the role of interleukin-6. Kidney international Supplement 2002 80 103 108 10.1046/j.1523-1755.61.s80.19.x 11982823

[B9] Copeland WE Shanahan L Worthman C Angold A Costello EJ Cumulative depression episodes predict later C-reactive protein levels: a prospective analysis Biol Psychiatry 2012 71 1 15 21 10.1016/j.biopsych.2011.09.023 22047718 PMC3586231

[B10] Hoang MT Defina LF Willis BL Leonard DS Weiner MF Brown ES Association between low serum 25-hydroxyvitamin D and depression in a large sample of healthy adults: the Cooper Center longitudinal study Mayo Clin Proc 2011 86 11 1050 1055 10.4065/mcp.2011.0208 22033249 PMC3202994

[B11] Brouwer-Brolsma EM Feskens EJ Steegenga WT de Groot LC Associations of 25-hydroxyvitamin D with fasting glucose, fasting insulin, dementia and depression in European elderly: the SENECA study Eur J Nutr 2013 52 3 917 925 10.1007/s00394-012-0399-0 22729969 PMC3611027

[B12] Tolppanen AM Sayers A Fraser WD Lewis G Zammit S Lawlor DA The association of serum 25-hydroxyvitamin D3 and D2 with depressive symptoms in childhood–a prospective cohort study J Child Psychol Psychiatry 2012 53 7 757 766 10.1111/j.1469-7610.2011.02518.x 22211693 PMC3412227

[B13] Singer RF Vitamin D, in dialysis: defining deficiency and rationale for supplementation Semin Dial 2013 26 1 40 46 10.1111/sdi.12010 23017052

[B14] Duranton F Rodriguez-Ortiz ME Duny Y Rodriguez M Daures JP Argiles A Vitamin D treatment and mortality in chronic kidney disease: a systematic review and meta-analysis Am J Nephrol 2013 37 3 239 248 10.1159/000346846 23467111

[B15] Metzger M Houillier P Gauci C Haymann JP Flamant M Thervet E Boffa JJ Vrtovsnik F Froissart M Stengel B Ureña-Torres P Relation between circulating levels of 25(OH) vitamin D and parathyroid hormone in chronic kidney disease: quest for a threshold J Clin Endocrinol Metab 2013 98 7 2922 2928 10.1210/jc.2013-1294 23633202

[B16] Bruchas RR de Las FL Carney RM Reagan JL Bernal-Mizrachi C Riek AE Gu CC Bierhals A Schootman M Malmstrom TK Burroughs TE Stein PK Miller DK Dávila-Román VG The St. Louis African American health-heart study: methodology for the study of cardiovascular disease and depression in young-old African Americans BMC Cardiovasc Disord 2013 13 66 10.1186/1471-2261-13-66 24011389 PMC3847628

[B17] Craven JL Rodin GM Littlefield C The Beck Depression Inventory as a screening device for major depression in renal dialysis patients Int J Psychiatry Med 1988 18 4 365 374 10.2190/M1TX-V1EJ-E43L-RKLF 3235282

[B18] Yeung A Howarth S Chan R Sonawalla S Nierenberg AA Fava M Use of the Chinese version of the Beck Depression Inventory for screening depression in primary care J Nerv Ment Dis 2002 190 2 94 99 10.1097/00005053-200202000-00005 11889362

[B19] Shek DT Depressive symptoms in alpha sample of chinese adolescents: an empirical study using the Chinese version of the beck depression inventory Int J Adolesc Med Health 1992 5 1 1 16 22912105 10.1515/IJAMH.1991.5.1.1

[B20] Zhang J Huang C Li Y Chen J Shen F Yao Q Qian J Bao B Yao X Health-related quality of life in dialysis patients with constipation: a cross-sectional study Patient Prefer Adherence 2013 7 589 594 23814466 10.2147/PPA.S45471 PMC3693922

[B21] Sezer S Uyar ME Bal Z Tutal E Ozdemir AFN The influence of socioeconomic factors on depression in maintenance hemodialysis patients and their caregivers Clin Nephrol 2013 80 11 342 348 10.5414/CN107742 24091317

[B22] Nowak L Adamczak M Wiecek A Is inflammation a new risk factor of depression in haemodialysis patients Int Urol Nephrol 2013 45 4 1121 1128 10.1007/s11255-012-0269-y 22972567 PMC3732758

[B23] Wu PC Huang TW Gender-related invariance of the Beck Depression Inventory II for Taiwanese adolescent samples Assessment 2012 2012 2012 10.1177/1073191112441243 22517921

[B24] Appelhans BM Whited MC Schneider KL Ma Y Oleski JL Merriam PA Waring ME Olendzki BC Mann DM Ockene IS Pagoto SL Depression severity, diet quality, and physical activity in women with obesity and depression J Acad Nutr Diet 2012 112 5 693 698 10.1016/j.jand.2012.02.006 22709773 PMC3378978

[B25] Klumpp H Post D Angstadt M Fitzgerald DA Phan KL Anterior cingulate cortex and insula response during indirect and direct processing of emotional faces in generalized social anxiety disorder Biol Mood Anxiety Disord 2013 3 1 7 10.1186/2045-5380-3-7 23547713 PMC3632493

[B26] Rebollo P Ortega F Baltar JM Badía X Alvarez-Ude F Díaz-Corte C Naves M Navascúes RA Ureña A Alvarez-Grande J Health related quality of life (HRQOL) of kidney transplanted patients: variables that influence it Clin Transplant 2000 14 3 199 207 10.1034/j.1399-0012.2000.140304.x 10831077

[B27] Zhang F Nunes M Segmuller B Dunphy R Hesse RH Setty SK Degradation chemistry of a Vitamin D analogue (ecalcidene) investigated by HPLC-MS, HPLC-NMR and chemical derivatization J Pharm Biomed Anal 2006 40 4 850 863 10.1016/j.jpba.2005.07.052 16242878

[B28] Jafri L Khan AH Siddiqui AA Mushtaq S Iqbal R Ghani F Siddiqui I Comparison of high performance liquid chromatography, radio immunoassay and electrochemiluminescence immunoassay for quantification of serum 25 hydroxy vitamin D Clin Biochem 2011 44 10–11 864 868 21570387 10.1016/j.clinbiochem.2011.04.020

[B29] Snellman G Melhus H Gedeborg R Olofsson S Wolk A Pedersen NL Michaëlsson K Seasonal genetic influence on serum 25-hydroxyvitamin D levels: a twin study PLoS One 2009 4 11 e7747 10.1371/journal.pone.0007747 19915719 PMC2774516

[B30] Nabolsi MM Wardam L Al-Halabi JO Quality of life, depression, adherence to treatment and illness perception of patients on haemodialysis Int J Nurs Pract 2013 doi:10.1111/ijn.12205 10.1111/ijn.12205 24124912

[B31] Preljevic VT Østhus TB Os I Sandvik L Opjordsmoen S Nordhus IH Dammen T Lower residual renal function is a risk factor for depression and impaired health-related quality of life in Korean peritoneal dialysis patients J Korean Med Sci 2012 27 1 64 71 10.3346/jkms.2012.27.1.64 22219616 PMC3247777

[B32] Khalil AA Frazier SK Depressive symptoms and dietary nonadherence in patients with end-stage renal disease receiving hemodialysis: a review of quantitative evidence Issues Ment Health Nurs 2010 31 5 324 330 10.3109/01612840903384008 20394478

[B33] Lew SQ Piraino B Quality of life and psychological issues in peritoneal dialysis patients Semin Dial 2005 18 2 119 123 15771655 10.1111/j.1525-139X.2005.18215.x

[B34] Ibrahim S El SO Depression, quality of life and malnutrition-inflammation scores in hemodialysis patients Am J Nephrol 2008 28 5 784 791 10.1159/000131101 18463431

[B35] Nanri A Mizoue T Matsushita Y Poudel-Tandukar K Sato M Ohta M Mishima N Association between serum 25-hydroxyvitamin D and depressive symptoms in Japanese: analysis by survey season Eur J Clin Nutr 2009 63 12 1444 1447 10.1038/ejcn.2009.96 19690578

[B36] Pan A Lu L Franco OH Yu Z Li H Lin X Association between depressive symptoms and 25-hydroxyvitamin D in middle-aged and elderly Chinese J Affect Disord 2009 118 1–3 240 243 19249103 10.1016/j.jad.2009.02.002

[B37] Brandenbarg J Vrijkotte TG Goedhart G van Eijsden M Maternal early-pregnancy vitamin D status is associated with maternal depressive symptoms in the Amsterdam Born Children and Their Development cohort Psychosom Med 2012 74 7 751 757 10.1097/PSY.0b013e3182639fdb 22879429

[B38] Cassidy-Bushrow AE Peters RM Johnson DA Li J Rao DS Vitamin D nutritional status and antenatal depressive symptoms in African American women J Women’s Health 2012 21 11 1189 1195 10.1089/jwh.2012.3528 22823176

[B39] Lee DM Tajar A O’Neill TW O'Connor DB Bartfai G Boonen S Bouillon R Casanueva FF Finn JD Forti G Giwercman A Han TS Huhtaniemi IT Kula K Lean ME Punab M Silman AJ Vanderschueren D Wu FC Pendleton N Lower vitamin D levels are associated with depression among community-dwelling European men J Psychopharmacol (Oxford, England) 2011 25 10 1320 1328 10.1177/0269881110379287 20823081

[B40] Penckofer S Kouba J Byrn M Estwing FC Vitamin D and depression: where is all the sunshine Issues Ment Health Nurs 2010 31 6 385 393 10.3109/01612840903437657 20450340 PMC2908269

[B41] Kjaergaard M Waterloo K Wang CE Almås B Figenschau Y Hutchinson MS Svartberg J Jorde R Effect of vitamin D supplement on depression scores in people with low levels of serum 25-hydroxyvitamin D: nested case–control study and randomised clinical trial Br J Psychiatry 2012 201 5 360 368 10.1192/bjp.bp.111.104349 22790678

[B42] Bertone-Johnson ER Powers SI Spangler L Larson J Michael YL Millen AE Bueche MN Salmoirago-Blotcher E Wassertheil-Smoller S Brunner RL Ockene I Ockene JK Liu S Manson JE Vitamin D supplementation and depression in the women’s health initiative calcium and vitamin D trial Am J Epidemiol 2012 176 1 1 13 10.1093/aje/kwr482 22573431 PMC3385159

[B43] Hogberg G Gustafsson SA Hallstrom T Gustafsson T Klawitter B Petersson M Depressed adolescents in a case-series were low in vitamin D and depression was ameliorated by vitamin D supplementation Acta Paediatrica (Oslo, Norway: 1992) 2012 101 7 779 783 10.1111/j.1651-2227.2012.02655.x 22372707

[B44] Balsamo M Imperatori C Sergi MR Belvederi Murri M Continisio M Tamburello A Innamorati M Saggino A Cognitive vulnerabilities and depression in young adults: An ROC curves analysis Depress Res Treat 2013 2013 407602 24058734 10.1155/2013/407602 PMC3766551

[B45] Pilz S Gaksch M O’Hartaigh B Tomaschitz A Marz W The role of vitamin D deficiency in cardiovascular disease: where do we stand in 2013 Arch Toxicol 2013 87 12 2083 2103 10.1007/s00204-013-1152-z 24173581

[B46] Messa P Curreri M Regalia A Alfieri CM Vitamin D and the cardiovascular system: an overview of the recent literature Am J Cardiovasc Drugs 2013 14 1 1 14 24122604 10.1007/s40256-013-0047-y

